# Supplementary material for: Variability in Breast Cancer Biomarker Assessment and the Effect on Oncological Treatment Decisions: A Nationwide 5-Year Population-Based Study
Source: Cancers (Basel). 2021 Mar 9;13(5):1166. doi: 10.3390/cancers13051166 (PMC7963154; doi:10.3390/cancers13051166)
Supplement: Supplementary file 1 [file cancers-13-01166-s001.pdf]

Supplementary Material: Variability in Breast Cancer Biomarker Assessment and the Effect on Oncological Treatment Decisions: A Nationwide 5-Year Population-Based Study

Balazs Acs, Irma Fredriksson, Caroline Rönnlund, Catharina Hagerling, Anna Ehinger, Anikó Kovács, Rasmus Røge, Jonas Bergh and Johan Hartman

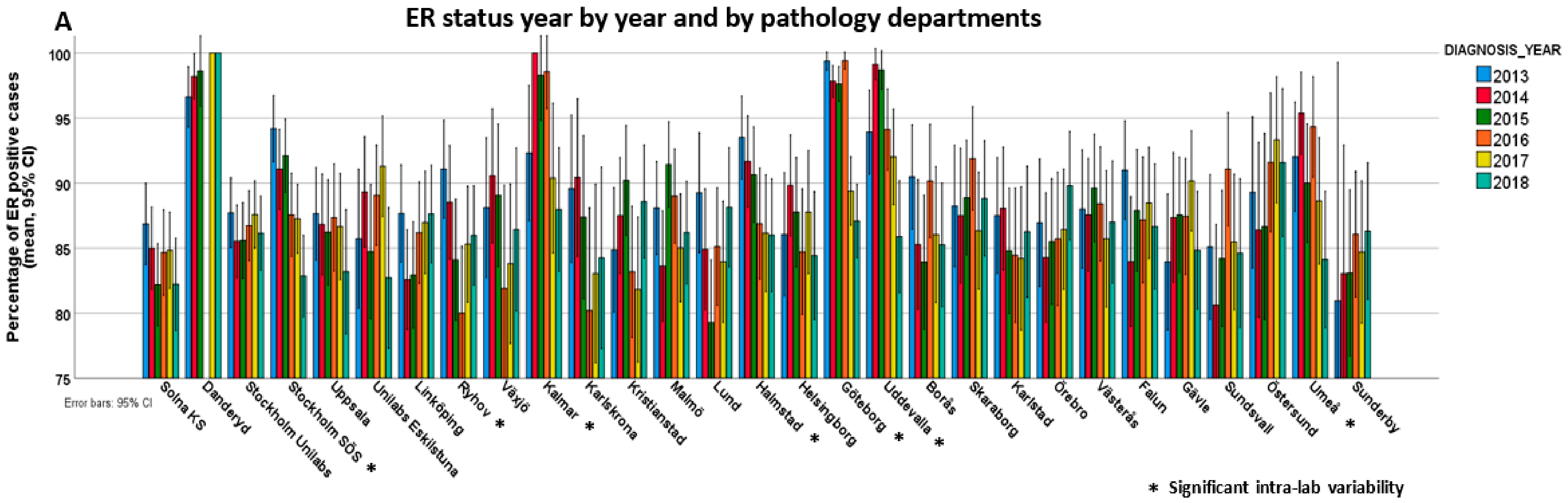
A
ER status year by year and by pathology departments
Percentage of ER positive cases (mean, 95% CI)
DIAGNOSIS_YEAR
2013
2014
2015
2016
2017
2018
Error bars: 95% CI
Solna KS
Danderyd
Stockholm Unilabs
Stockholm SÖS *
Uppsala
Unilabs Eskilstuna
Linköping
Ryhov *
Växjö
Kalmar *
Karlskrona
Kristianstad
Malmö
Lund
Halmstad *
Helsingborg
Göteborg *
Uddevalla *
Borås
Skaraborg
Karlstad
Örebro
Västerås
Falun
Gävle
Sundsvall
Östersund
Umeå *
Sunderby
* Significant intra-lab variability

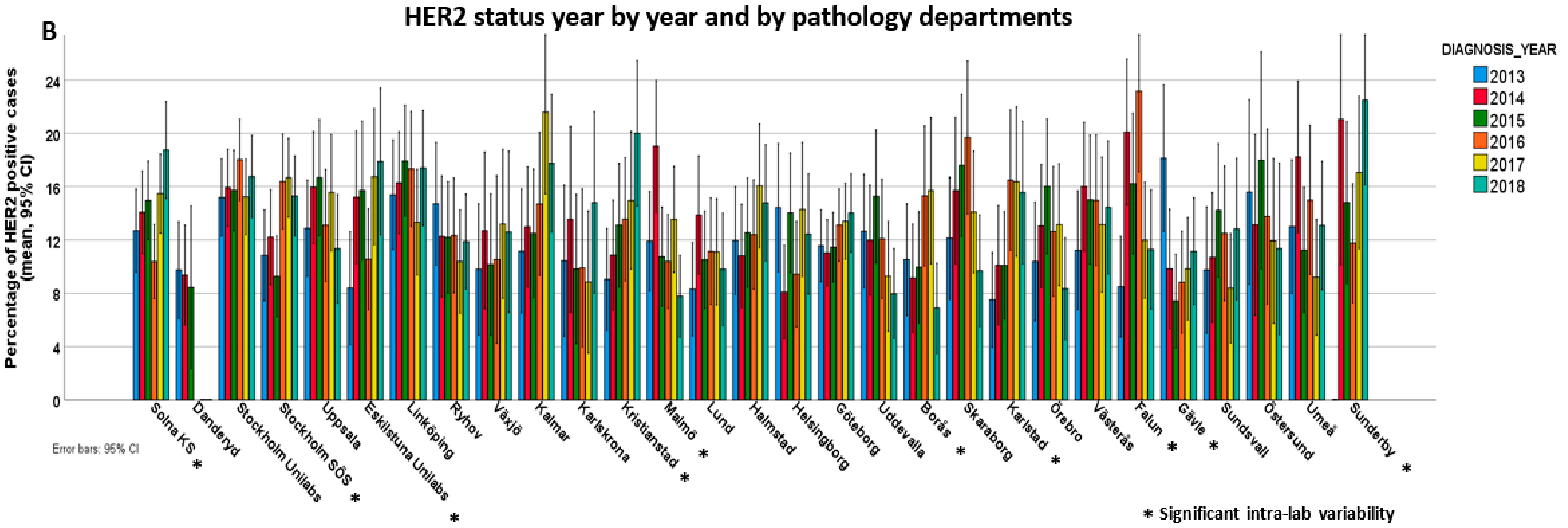
B
HER2 status year by year and by pathology departments
Percentage of HER2 positive cases (mean, 95% CI)
DIAGNOSIS_YEAR
2013
2014
2015
2016
2017
2018
Error bars: 95% CI
Solna KS
Danderyd *
Stockholm Unilabs
Stockholm SÖS *
Uppsala
Eskilstuna Unilabs *
Linköping
Ryhov
Växjö
Kalmar
Karlskrona
Kristianstad *
Malmö *
Lund
Halmstad
Helsingborg
Göteborg
Uddevalla
Borås *
Skaraborg
Karlstad *
Örebro
Västerås
Falun *
Gävle *
Sundsvall
Östersund
Umeå
Sunderby *
* Significant intra-lab variability

Figure S1. Intra-lab variability for estrogen receptor (ER) (**A**) and human epidermal growth-factor receptor 2 (HER2) (**B**) status among pathology departments. Lab Danderyd had very few cases in recent years because the lab was shutting down for breast cancer diagnostics. These cases were excluded from the statistical analyses.

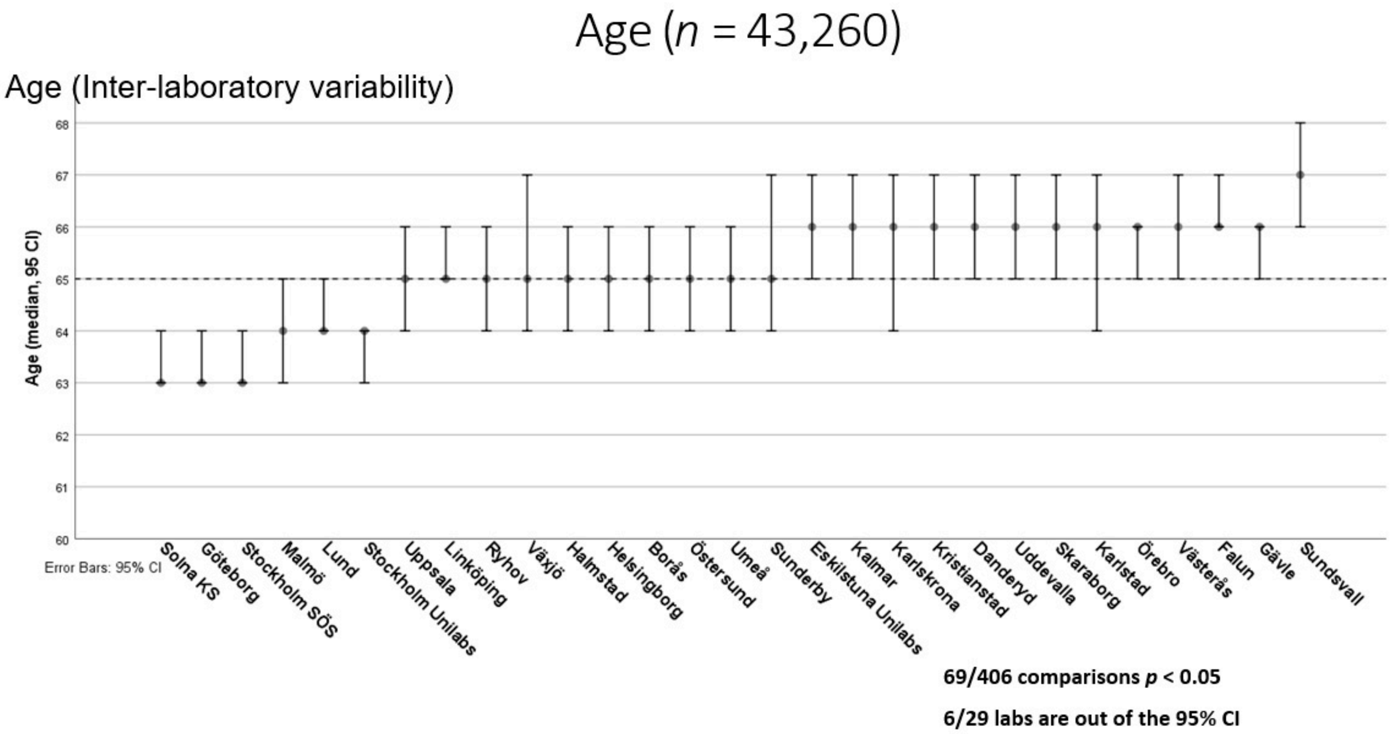

T size (mm) (n = 37,649)

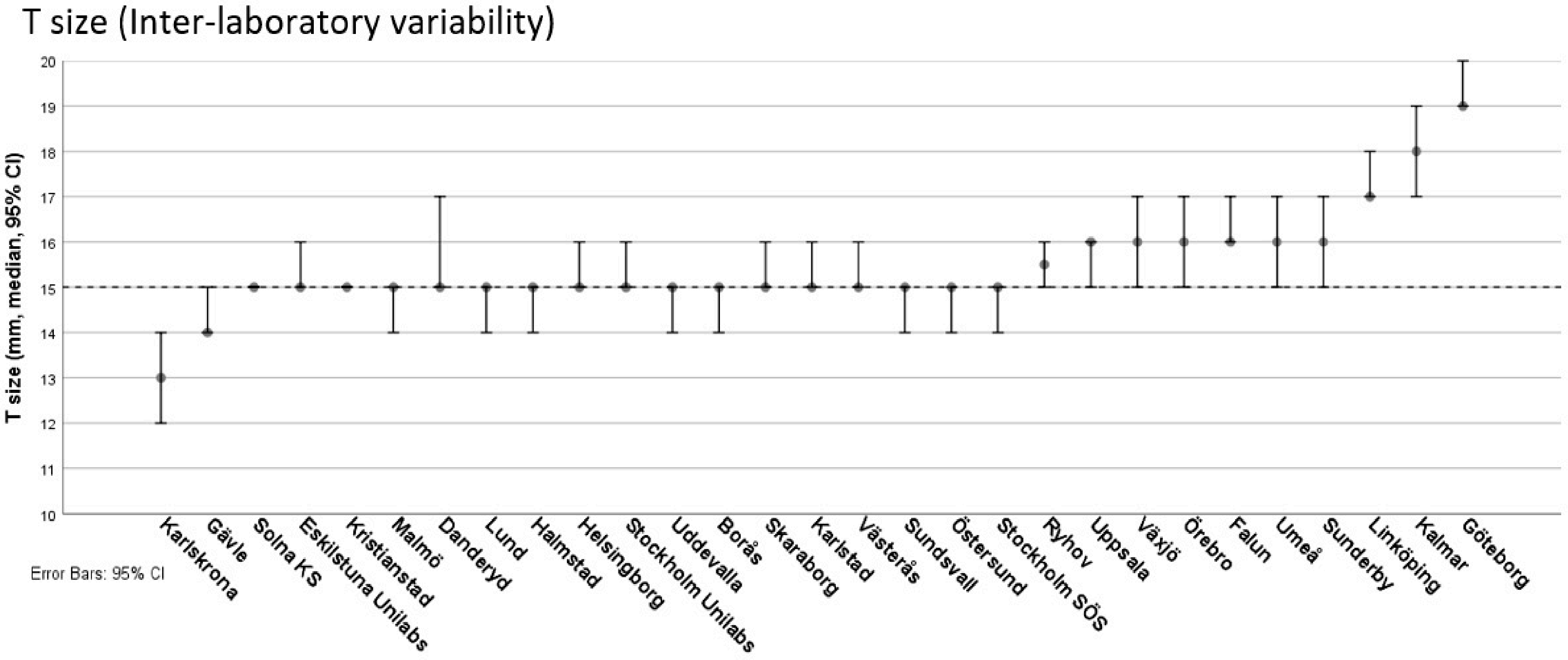

5/29 labs are outside of the 95% CI

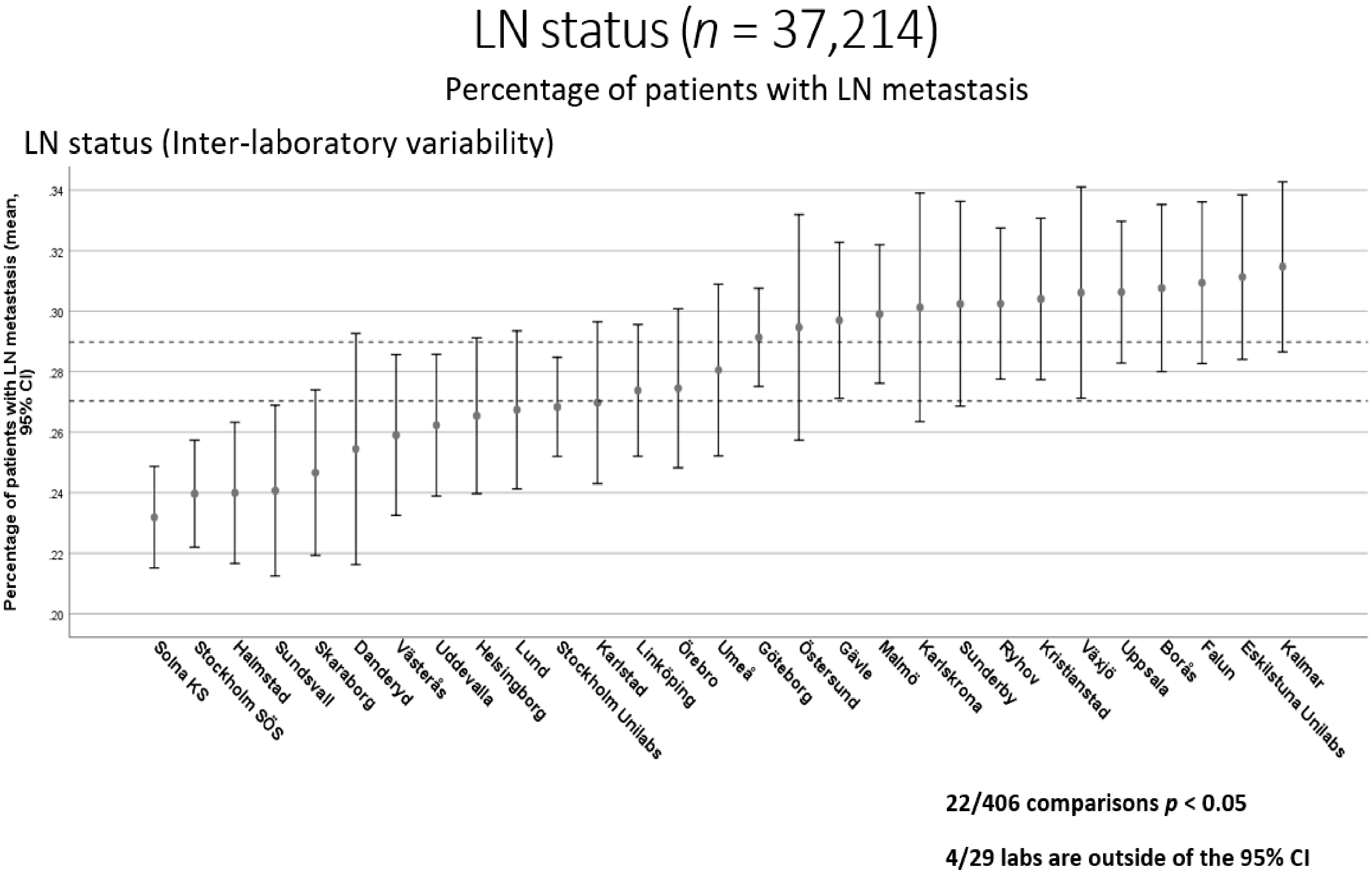

Figure S2. Distribution of Age, T size, and LN status.

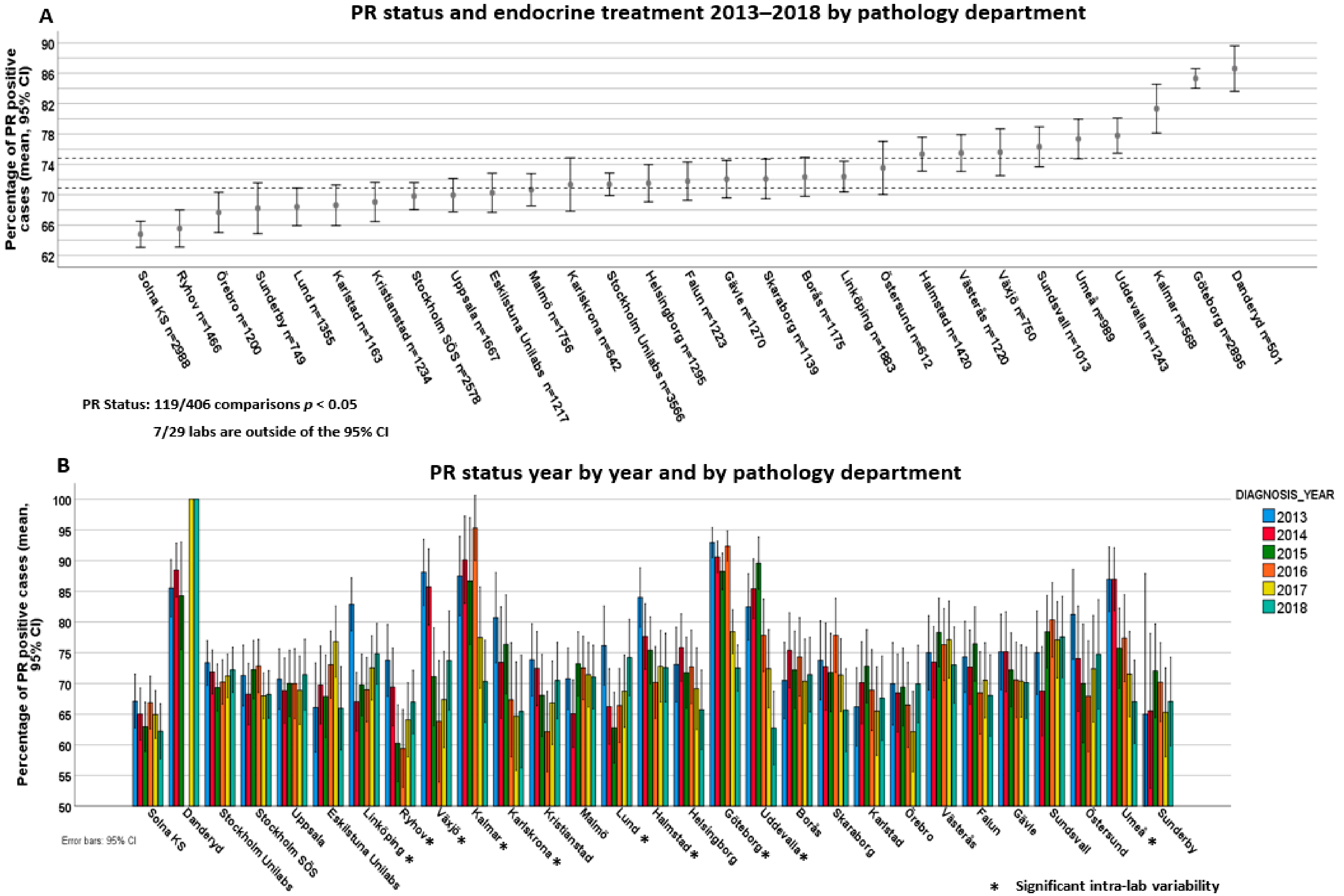
A
PR status and endocrine treatment 2013–2018 by pathology department
Percentage of PR positive cases (mean, 95% CI)
90
86
82
78
74
70
66
62
Solna KS n=2988
Ryhov n=1466
Örebro n=1200
Sunderby n=749
Lund n=1355
Karlstad n=1163
Kristianstad n=1234
Stockholm SÖS n=2578
Uppsala n=1667
Eskilstuna Unilabs n=1217
Malmö n=1756
Karlskrona n=642
Stockholm Unilabs n=3566
Helsingborg n=1295
Falun n=1223
Gävle n=1270
Skaraborg n=1139
Borås n=1175
Linköping n=1883
Östersund n=612
Halmstad n=1420
Västerås n=1220
Växjö n=750
Sundsvall n=1013
Umeå n=989
Uddevalla n=1243
Kalmar n=568
Göteborg n=2895
Danderyd n=501
PR Status: 119/406 comparisons p < 0.05
7/29 labs are outside of the 95% CI
B
PR status year by year and by pathology department
Percentage of PR positive cases (mean, 95% CI)
100
95
90
85
80
75
70
65
60
55
50
DIAGNOSIS_YEAR
2013
2014
2015
2016
2017
2018
Solna KS
Danderyd
Stockholm Unilabs
Stockholm SÖS
Uppsala
Eskilstuna Unilabs
Linköping *
Ryhov *
Växjö *
Kalmar *
Karlskrona *
Kristianstad
Malmö
Lund *
Halmstad *
Helsingborg
Göteborg *
Uddevalla *
Borås
Skaraborg
Karlstad
Örebro
Västerås
Falun
Gävle
Sundsvall
Östersund
Umeå *
Sunderby
Error bars: 95% CI
* Significant intra-lab variability

Figure S3. Inter- (**A**) and intra-laboratory variability (**B**) for progesterone receptor (PR) status among 29 pathology departments.

Table S1. NordiQC—Sweden—Mamma module—extended data.

**Assessment Marks for Breast Cancer**	**O**	**G**	**B**	**P**
Estrogen receptor	23	5	1	0
HER2 IHC	25	1	1	0
Progesterone receptor	20	7	1	0
Platforms (due to confidentiality, platforms with less than 3 users are excluded)				
Estrogen Receptor				
Dako Omnis	6	0	0	0
Ventana Benchmark Ultra	14	4	1	0
HER2 IHC				
Dako Autostainer Link 48 +	4	0	0	0
Ventana Benchmark Ultra	18	1	0	0
Progesterone Receptor				
Dako Omnis	4	2	0	0
Ventana Benchmark Ultra	15	3	1	0
Clones (due to confidentiality, clones with less than 2 users are excluded)				
Estrogen Receptor				
6F11	1	1	0	0
EP1	7	0	0	0
SP1	15	4	1	0
HER2 IHC				
4B5	3	0	0	0
HercepTest (polyclonal)	4	0	0	0
PATHWAY (clone 4B5)	17	1	0	0
Progesterone Receptor				
16	0	1	1	0
1E2	15	2	0	0
PR 1294	4	2	0	0
PR 636	1	1	0	0

This report summarizes the latest assessment marks from Sweden in the breast cancer module B29. O = Optimal, G = Good, B = Borderline, P = Poor, # Overall. For more details about NordiQC Breast cancer module B29, please follow this link (https://www.nordiqc.org/modules.php)./RR - 2020.09.13.

Table S2. Quality assurance of NKBC data.

Biomarker	Error Rate	Systematic Check
	All data n = 1076	
Grade	2.97%	27 mismatch and 5 missing data
ER % score	2.32%	21 mismatch and 4 missing data
ER status	1.20%	3 mismatch and 10 missing data
PR % score	5.01%	42 mismatch and 12 missing data
PR status	2.88%	14 mismatch and 17 missing data
HER2 IHC status *	2.32%	17 mismatch and 8 missing data
HER2 ISH status **	1.57%	3 mismatch and 14 missing data
Ki67 % score	3.62%	27 mismatch and 12 missing data
Ki67 category status	25.27%	112 mismatch and 2 missing data
	Stockholm SÖS n = 144	
Grade	3.47%	3 mismatch and 2 missing data
ER % score	2.08%	2 mismatch and 1 missing data
ER status	1.38%	1 mismatch and 1 missing data
PR % score	5.55%	7 mismatch and 1 missing data
PR status	2.08%	2 mismatch and 1 missing data
HER2 IHC status ***	0.69%	1 mismatch
HER2 ISH status †	0.69%	1 mismatch
Ki67 % score	9.03%	12 mismatch and 1 missing data
Ki67 category status	31.25%	44 mismatch and 1 missing data
	Solna KS n = 144	
Grade	5.55%	7 mismatch and 1 missing data
ER % score	3.47%	4 mismatch and 1 missing data
ER status	1.38%	2 missing data
PR % score	8.33%	11 mismatch and 1 missing data
PR status	4.86%	5 mismatch and 2 missing data
HER2 IHC status ††	2.77%	4 mismatch
HER2 ISH status †††	0.69%	1 mismatch
Ki67 % score	4.86%	6 mismatch and 1 missing data
Ki67 category status	27.77%	39 mismatch and 1 missing data
	Danderyd n = 68	
Grade	7.35%	5 mismatch
ER % score	0%	
ER status	1.47%	1 missing data

PR % score	1.47%	1 missing data
PR status	4.41%	3 mismatch
HER2 IHC status [$]	1.47%	1 mismatch
HER2 ISH status [$$]	0%	
Ki67 % score	2.94%	1 mismatch and 1 missing data
Ki67 category status	35.29%	24 mismatch
	Stockholm region *n* = 356	
Grade	5.05%	15 mismatch and 3 missing data
ER % score	2.24%	6 mismatch and 2 missing data
ER status	1.40%	2 mismatch and 3 missing data
PR % score	5.89%	18 mismatch and 3 missing data
PR status	3.65%	10 mismatch and 3 missing data
HER2 IHC status [$$$]	1.68%	6 mismatch
HER2 ISH status [a]	0.56%	2 mismatch
Ki67 % score	6.17%	19 mismatch and 3 missing data
Ki67 category status	30.61%	107 mismatch and 2 missing data
	Lund *n* = 144	
Grade	0.69%	1 mismatch
ER % score	5.55%	1 mismatch and 7 missing data
ER status	2.77%	4 missing data
PR % score	8.33%	11 mismatch and 1 missing data
PR status	7.63%	2 mismatch and 9 missing data
HER2 IHC status [b]	4.16%	4 mismatch and 2 missing data
HER2 ISH status [c]	2.08%	3 missing data
Ki67 % score	4.16%	2 mismatch and 4 missing data
Ki67 category status	Not available	
	Malmö *n* = 144	
Grade	1.38%	2 mismatch
ER % score	0.69%	1 missing data
ER status	0.69%	1 missing data
PR % score	3.47%	4 mismatch and 1 missing data
PR status	2.08%	2 mismatch and 1 missing data
HER2 IHC status [d]	2.08%	3 mismatch
HER2 ISH status [e]	3.47%	1 mismatch and 4 missing data
Ki67 % score	2.08%	2 mismatch and 2 missing data
Ki67 category status	Not available	

	Kristianstad n = 144	
Grade	1.38%	1 mismatch and 1 missing data
ER % score	0%	
ER status	1.38%	2 missing data
PR % score	4.16%	6 mismatch
PR status	1.38%	2 missing data
HER2 IHC status [f]	0.69%	1 mismatch
HER2 ISH status [g]	2.77%	4 missing data
Ki67 % score	0.69%	1 mismatch
Ki67 category status	Not available	
	Helsingborg n = 144	
Grade	4.16%	5 mismatch and 1 missing data
ER % score	2.08%	3 mismatch
ER status	0.69%	1 mismatch
PR % score	2.08%	3 mismatch
PR status	0%	
HER2 IHC status [h]	4.16%	3 mismatch and 3 missing data
HER2 ISH status [i]	2.08%	3 missing data
Ki67 % score	1.38%	1 mismatch and 1 missing data
Ki67 category status	3.47%	5 mismatch (but 49 cases were missing)
	Skåne region n = 576	
Grade	1.90%	9 mismatch and 2 missing data
ER % score	2.08%	10 mismatch and 2 missing data
ER status	1.38%	1 mismatch and 7 missing data
PR % score	4.51%	18 mismatch and 8 missing data
PR status	2.77%	4 mismatch and 12 missing data
HER2 IHC status [j]	2.77%	8 mismatch and 8 missing data
HER2 ISH status [k]	2.60%	1 mismatch and 14 missing data
Ki67 % score	2.08%	6 mismatch and 6 missing data
Ki67 category status	only available in Helsingborg	
	Göteborg n = 144	
Grade	2.08%	3 mismatch
ER % score	3.47%	5 mismatch
ER status	0%	
PR % score	4.86%	6 mismatch and 1 missing data
PR status	1.38%	2 missing data

HER2 IHC status [l]	2.08%	3 mismatch
HER2 ISH status [m]	0%	
Ki67 % score	4.16%	3 mismatch and 3 missing data
Ki67 category status	Not available	

We selected 8 pathology departments in 3 regions to participate in the QC: Stockholm SÖS, Solna KS, Danderyd—Stockholm region; Lund, Malmö, Kristianstad, Helsingborg—Skåne region; Göteborg. Systematic check: 2 cases from every month between 2013 and 2018 at every site were randomly selected to retrieve pathology report: 144 cases per hospital; DS had cases until 2015 October→ 68 cases; NKBC data was compared to original pathology report in a total of 1076 breast cancer cases; Data delivery with encrypted and password protected files. Ki67 Category status: Stockholm region: Reported according to the current Ki67 cut-offs; Skåne region: In QC only reported in Helsingborg.* No data in NKBC on HER2 IHC until December 2014 → 369 cases missing; ** More ISH results in NKBC than in pathology reports: 460 cases with ISH in NKBC but without so in reports; *** No data in NKBC on HER2 IHC until December 2014 → 47 cases missing; [†] More ISH results in NKBC than in SÖS pathology reports: 75 cases; [††] No data in NKBC on HER2 IHC until December 2014 → 48 cases missing; [†††] More ISH results in NKBC than in KS pathology reports: 65 cases; [$] No data in NKBC on HER2 IHC until December 2014 → 44 cases missing; [$$] More ISH results in NKBC than in DS pathology reports: 50 cases; [$$$] No data in NKBC on HER2 IHC until December 2014 → 138 cases missing; [a] More ISH results in NKBC than in pathology reports: 190 cases with ISH in NKBC but without so in reports; [b] No data in NKBC on HER2 IHC until December 2014 → 47 cases missing; [c] More ISH results in NKBC than in Lund pathology reports: 39 cases; [d] No data in NKBC on HER2 IHC until December 2014 → 45 cases missing; [e] More ISH results in NKBC than in Malmö pathology reports: 33 cases; [f] No data in NKBC on HER2 IHC until December 2014 → 45 cases missing; [g] More ISH results in NKBC than in Kristianstad pathology reports: 39 cases; [h] No data in NKBC on HER2 IHC until December 2014 → 47 cases missing; [i] More ISH results in NKBC than in Kristianstad pathology reports: 33 cases; [j] No data in NKBC on HER2 IHC until December 2014 → 184 cases missing; [k] More ISH results in NKBC than in pathology reports: 144 cases with ISH in NKBC but without so in reports; [l] No data in NKBC on HER2 IHC until December 2014 → 47 cases missing; [m] More ISH results in NKBC than in Göteborg pathology reports: 126 cases.

Table S3. Questionnaire on analytical procedures.

Variables		Stockholm SÖS	Solna KS	Danderyd	Göteborg	Kristianstad	Lund	Malmö	Helsingborg
Type of Hematoxylin–Eosin (HE)/Staining platform		Leica HistoCore SPECTRA Workstation with HE Kit 3801654	Leica HistoCore SPECTRA Workstation with HE Kit 3801654	Leica HistoCore SPECTRA Workstation with HE Kit 3801654	Histolab and Dako	Hematoxylin, CS700, Eosin CS701 with Dako Coverstainer	Dako Sara I with Dako HE	Dako Sara I with Dako HE	Hematoxylin (Histolab), Eosin (Merck) with Sakuras Tissue Tek Prisma
Immunohistochemistry	ER	Clone SP1 (Roche 790-4324)	Clone SP1 (Roche 790-4324)	Clone SP1 (Roche 790-4324)	SP1 (Dako)	Clone SP1 (Roche 790-4324)	Clone SP1 (Roche 790-4324)	Clone SP1 (Roche 790-4324)	Clone SP1 (Roche 790-4324)
	PR	Clone 1E2 (Roche 790-2223)	Clone 1E2 (Roche 790-2223)	Clone 1E2 (Roche 790-2223)	636 (Dako)	Clone 1E2 (Roche 790-2223)	Clone 1E2 (Roche 790-2223)	Clone 1E2 (Roche 790-2223)	Clone 1E2 (Roche 790-2223)
	HER2	Clone 4B5 (Roche 790-2991)	Clone 4B5 (Roche 790-2991)	Clone 4B5 (Roche 790-2991)	HercepTest (DAKO)	Clone 4B5 (Roche 790-2991)	Clone 4B5 (Roche 790-2991)	Clone 4B5 (Roche 790-2991)	Clone 4B5 (Roche 790-2991)
	Ki67	Clone 30-9 (Roche 790-4286)	Clone 30-9 (Roche 790-4286)	Clone 30-9 (Roche 790-4286)	MIB-1 (Dako)	MIB-1 (Dako)	MIB-1 (Agilent)	MIB-1 (Agilent)	MIB-1 (Dako)

Platform	Ventana Benchmark Ultra (Roche)	Ventana Benchmark Ultra (Roche)	Ventana Benchmark Ultra (Roche)	Autostainer Link48 (DAKO)	Ventana Benchmark Ultra (Roche)	Ventana Benchmark Ultra (Roche)	Ventana Benchmark Ultra (Roche)	Ventana Benchmark Ultra (Roche)
HER2 in situ hybridization (ISH or SISH)	Roche/Ventana SISH Inform Her-2 DUAL ISH	Roche/Ventana SISH Inform Her-2 DUAL ISH	Roche/Ventana SISH Inform Her-2 DUAL ISH	SISH-test on Ventana Benchmark	Roche/Ventana SISH Inform Her-2 DUAL ISH	FISH with Ventana HER2 GenProtein (Roche)	Roche/Ventana SISH Inform Her-2 DUAL ISH	FISH with Ventana HER2 GenProtein (Roche)
Changed antibody or platform in the last 7 years?	No	No	No	No	No	No	No	No
Histological grading performed on digitized slides?	No	No	No	No	No	No but digital mitosis counting	Yes	Yes
Ki67 scoring with digital-image analysis?	Minimally	Minimally	Minimally	No	Yes	Yes	Yes	Yes

Leica, Wetzlar, Germany; Roche, Basel, Switzerland; Dako Agilent, Santa Clara, CA, United States; Histolab, Askim, Sweden; Merck, Kenilworth, NJ, United States.
